# Polymorphisms in DNA Repair Genes and *MDR1* and the Risk for Non-Hodgkin Lymphoma

**DOI:** 10.3390/ijms15046703

**Published:** 2014-04-21

**Authors:** Hee Nam Kim, Nan Young Kim, Li Yu, Yeo-Kyeoung Kim, Il-Kwon Lee, Deok-Hwan Yang, Je-Jung Lee, Min-Ho Shin, Kyeong-Soo Park, Jin-Su Choi, Hyeoung-Joon Kim

**Affiliations:** 1 Center for Creative Biomedical Scientists, Chonnam National University, Gwangju 501-746, Korea; E-Mails: kimhnamx@paran.com (H.N.K.); mhshinx@paran.com (M.-H.S.); 2 Environmental Health Center for Childhood Leukemia and Cancer, Chonnam National University Hwasun Hospital, Jeollanamdo 519-763, Korea; E-Mails: sylvia-plath@hanmail.net (N.Y.K.); cookie1018@nate.com (L.Y.); biospace@cnuh.com (I.-K.L.); 3 Department of Hematology/Oncology, Chonnam National University Hwasun Hospital 160 Ilsim-ri, Hwasun-eup, Hwasun-gun, Jellanam-do 519-809, Korea; E-Mails: elysium1015@hanmail.net (Y.-K.K.); drydh1685@hotmail.com (D.-H.Y.); drjejung@chonnam.ac.kr (J.-J.L.); 4 Department of Preventive Medicine, Chonnam National University Medical School, Gwangju 501-746, Korea; E-Mail: jschoix@chonnam.ac.kr; 5 Department of Preventive Medicine, College of Medicine, Seonam University, Namwon 590-711, Korea; E-Mail: pys0903@hitel.net

**Keywords:** NHL, polymorphism, association, DNA repair, *MDR1*

## Abstract

The damage caused by oxidative stress and exposure to cigarette smoke and alcohol necessitate DNA damage repair and transport by multidrug resistance-1 (MDR1). To explore the association between polymorphisms in these genes and non-Hodgkin lymphoma risk, we analyzed 15 polymorphisms of 12 genes in a population-based study in Korea (694 cases and 1700 controls). Four genotypes of DNA repair pathway genes (*XRCC1* 399 GA, *OGG1* 326 GG, *BRCA1* 871 TT, and *WRN* 787 TT) were associated with a decreased risk for NHL [odds ratio (*OR*)_XRCC1 GA_ = 0.80, *p* = 0.02; *OR*_OGG1 GG_ = 0.70, *p* = 0.008; *OR*_BRCA1 TT_ = 0.71, *p* = 0.048; *OR*_WRN TT_ = 0.68, *p* = 0.01]. Conversely, the *MGMT* 115 CT genotype was associated with an increased risk for NHL (*OR* = 1.25, *p* = 0.04). In the *MDR1* gene, the 1236 CC genotype was associated with a decreased risk for NHL (*OR* = 0.74, *p* = 0.04), and the 3435 CT and TT genotypes were associated with an increased risk (*OR*_3435CT_ = 1.50, *p* < 0.0001; *OR*_3435TT_ = 1.43, *p* = 0.02). These results suggest that polymorphisms in the DNA repair genes *XRCC1*, *OGG1*, *BRCA1*, *WRN1*, and *MGMT* and in the *MDR1* gene may affect the risk for NHL in Korean patients.

## 1. Introduction

The occurrence of non-Hodgkin lymphoma (NHL) has increased steadily over the last few decades. Environmental exposure, such as tobacco, alcohol consumption and diet, family history, immune dysfunction, immune stimulation, and infection, have all been associated with the risk for NHL [1–5]. In addition, many studies have suggested that genetic variation also plays a role in the pathogenesis of NHL [6–10].

DNA damage induced by oxidative stress and exposure to environmental chemical carcinogens such as smoking and benzene is repaired via DNA repair pathways. Polymorphisms in genes encoding these pathways can decrease or increase DNA repair activity and subsequently, contribute to the progression of various cancers [11].

DNA repair plays a major role in maintaining genomic stability via several pathways. The base excision repair (BER) pathway repairs damaged bases or single-strand DNA breaks; nucleotide excision repair (NER) excises a variety of helix-distorting DNA lesions; and homologous recombination (HR) and non-homologous end joining (NHEJ) repair double-strand breaks and direct damage reversal. Several polymorphisms identified in genes of the BER pathway (*XRCC1* and *OGG1*), NER pathway (*ERCC1*, *XPC*, and *ERCC2*/*XPD*), HR pathway (*RAD51*, *BRCA1*, *XRCC3*, and *WRN*), and NHEJ pathway (*XRCC4* and *MGMT*) have been shown to be associated with risks for various cancers [7,10,12–15].

P-glycoprotein (P-gp), which is encoded by the human multidrug resistance-1 gene (*MDR1*, *ABCB1*), has a protective effect in cells [16,17]. As an ATP-dependent drug efflux pump, P-gp transports a wide variety of lipophilic compounds from inside to outside the cell [18–20]. Two synonymous polymorphisms, C1236T and C3435T, and one nonsynonymous polymorphism, G2677T/A, are the most common polymorphisms in the coding region of *MDR1*. These polymorphisms are associated with increased *MDR1* mRNA expression levels [21–24].

Although several studies have reported associations between some polymorphisms in these genes and the risk for NHL [10,25–30], the results have not been consistent. To our knowledge there has been no report describing an association between *MDR1* polymorphisms and the risk for NHL. In the present study, we investigated whether polymorphisms in DNA repair genes or *MDR1* are associated with risk for 694 NHL and 378 diffuse large B-cell lymphoma (DLBCL) and 140 T-cell lymphoma subtype, by analyzing 15 polymorphisms in 12 genes (Table 1).

## 2. Results

The distribution of polymorphisms in the DNA repair genes and *MDR1* (*ABCB1*) among the NHL cases and the controls are shown in Tables 2 and 3. The control genotype frequencies were within Hardy–Weinberg equilibrium and did not significantly differ between men and women (data not shown). The genotype frequencies of these polymorphisms except *RAD51* rs1801321 by us were similar to HapMap project. There is no report of *RAD51* rs1801321 in Asian population in HapMap project and the SNP500Cancer Database.

Among the DNA repair genes, the polymorphism genotypes *XRCC1* 399 GA and *WRN* 787 TT were associated with a decreased risk for NHL (odds ratio (*OR*)_XRCC1 GA_ = 0.80, *p* = 0.02; *OR*_WRN 787 TT_ = 0.68, *p* = 0.01). The *OGG1* 326 GG and *BRCA1* 871 TT genotypes were associated with a decreased risk for NHL (*OR*_OGG1 GG_ = 0.70, *p* = 0.008; *OR*_BRCA1 TT_ = 0.71, *p* = 0.048), DLBCL (*OR*_OGG1 GG_ = 0.68, *p* = 0.02; *OR*_BRCA1 TT_ = 0.62, *p* = 0.04), and T-cell lymphoma (*OR*_OGG1 GG_ = 0.59, *p* = 0.04; *OR*_BRCA1 TT_ = 0.42, *p* = 0.04). Conversely, the *MGMT* 115 CT genotype was associated with an increased risk for NHL (*OR* = 1.25, *p* = 0.04) and DLBCL (*OR* = 1.37, *p* = 0.02).

For *ABCB1* polymorphisms, the 1236 CC genotype was associated with a decreased risk for NHL (*OR* = 0.74, *p* = 0.04) and DLBCL (*OR* = 0.64, *p* = 0.02), whereas the 3435 CT genotype was associated with an increased risk for NHL (*OR* = 1.50, *p* < 0.0001), DLBCL (*OR* = 1.52, *p* = 0.01), and T-cell lymphoma (*OR* = 1.51, *p* = 0.03). Using subjects with the T-T-T haplotype of *ABCB1* 1236, 2677, and 3435 as a reference group, the C-G-C haplotype was associated with a decreased risk for NHL (*OR* = 0.83, *p* = 0.04) and DLBCL (*OR* = 0.77, *p* = 0.03), whereas the C-G-T haplotype was associated with an increased risk for NHL (*OR* = 1.67, *p* = 0.02) and T-cell lymphoma (*OR* = 2.48, *p* = 0.007). No association was found between any other polymorphism evaluated in the present study and the risk for NHL.

## 3. Discussion

In the present population-based case-control study, we investigated whether genetic polymorphisms in DNA repair genes and *MDR1* were associated with the risk for developing NHL in a Korean population. For the DNA repair gene polymorphisms, the *XRCC1* 399 GA, *OGG1* 326 GG, *BRCA1* 871 TT, *WRN* 787 TT, and *MGMT* 115 CT genotypes were associated with the risk for NHL, as were the *ABCB1* 1236 CC and 3435 TT genotypes. The C-G-C, C-G-T, and T-G-T haplotypes of *ABCB1* 1236, 2677, and 3435 were significantly associated.

XRCC1, a central scaffolding protein for DNA ligase III [35], and OGG1, which encodes a DNA glycosylase that catalyzes the removal of 8-OH-Gua adducts [36,37], have been implicated in the BER system for the repair of damaged bases or single-strand DNA breaks. Previous studies suggest that XRCC1 Arg399Gln has been associated with higher levels of DNA adducts, and sister chromatid exchanges [12] and, thus, may lead to carcinogenesis. However, conversely with this hypothesis, our results showed that XRCC1 399 GA genotype was associated with a decreased risk for NHL, which is similar to a previous report from Australia [28]. The OGG1 326 GG genotype was associated with a decreased risk for NHL and DLBCL and T-cell subtype in this study. There is various evidence for OGG1 326Cys functional effect, such as lower DNA repair activity and the catalytic efficiency compared with 326Ser [38–40], or no difference in enzyme activity [41].

WRN and BRCA1 in the HR pathway are involved in recombination and the repair of double-strand breaks. Our result showed that the *WRN* 787 TT genotype was associated with a decreased risk of only NHL, and NHL including DLBCL and T-cell lymphoma for *BRCA1* 871 TT genotype. The WRN Leu787Leu polymorphism is a synonymous SNP and lies in the helicase domain of the WRN protein. The function of these polymorphisms is still unknown. Therefore, the biological function of these polymorphisms for the responsible effect on gene expression and linkage disequilibrium with the functional SNP should be investigated.

The product of MGMT (also known as AGT or ATase) in the DR pathway reverses *O*^6^-alkylation damage in DNA by transferring the alkyl group to a cysteine residue in the enzyme [42,43]. In the present study, MGMT 115 CT genotype was associated with a decreased risk for NHL and DLBCL. This finding is similar to that of Shen *et al.* [28]. The function of codon 115 in the *MGMT* gene is unknown [44,45], but, the possible effect of DNA guanine *O*^6^-alkylation damage by Leu115Phe may be due to a linkage between this variant and other functional variants [46,47].

Inconsistencies between our results and those of previous studies may be attributable to different frequencies of polymorphisms in different ethnicities, differences in sample sizes, or differences in environmental exposure.

Our result showed that *ABCB1* 1236 CC genotype was associated with a decreased risk, but, in contrast, *ABCB1* 3435 CT genotype was associated with an increased risk for NHL and DLBCL. The C-G-C haplotype was associated with a decreased risk for NHL and DLBCL, whereas the C-G-T haplotype was associated with an increased risk for NHL and T-cell lymphoma. Function of the C1236T was still unknown, while various effects of the *ABCB1* C3435T and G2677T/A polymorphisms have been observed, such as increased mRNA levels or no effect [21–24], silent C3435T polymorphism-altered P-gp activity [48–51], and reduced activity [52]. Several reports have suggested that these results may due to linkage disequilibrium with the 3435 and 2677 polymorphisms [24,33], allele-specific differences in mRNA folding [53,54], or numerous environmental factors [55].

However, this study has some imitations. These selected SNPs could not represent each gene, and functional analysis of these polymorphisms was not fully characterized. Moreover, the environmental factors were not considered. Due to the limitation of the power to detect associations, multiple comparisons were not considered in the present analysis. Thus, although some genotypes showed associations, there may be some false-positive associations. Future epidemiological studies with larger sample sizes stratified by other environmental factors such as lifestyle and chemical exposure are also necessary to confirm our findings.

## 4. Experimental Section

### 4.1. Study Population

The patients enrolled in this study were adults aged ≥15 years who had been diagnosed with NHL at Chonnam National University Hospital, Hwasun-gun, Korea between January 1997 and June 2008. There were 694 NHL cases, consisting of 420 men and 274 women, with a mean age of 55.4 ± 14.8 years (range, 15–89 years). 694 NHL cases consisted of 378 DLBCL, 140 T-cell lymphomas and 176 other lymphomas. 378 DLBCL were included only diffuse large B-cell lymphoma. 140 T-cell lymphomas consisted of peripheral T-cell lymphoma-unspecified, extranodal natural killer cell/T-cell lymphoma, anaplastic large cell lymphoma, angioimmunoblastic T-cell lymphoma, T-cell type lymphoblastic lymphoma and cutaneous T-cell NHL. 176 other lymphomas were included all other subtypes including follicular lymphoma, except Hodgkin disease.

The control group (*n* = 1700) was randomly selected from the general population in the same geographical areas as the cases and was composed of participants in the Thyroid Disease Prevalence Study, which was performed in the Yeonggwang and Muan counties of Jeollanam-do Province and Namwon City of Jeollabuk-do Province, Korea, from July 2004 to January 2006. A total of 4018 subjects (mean age, 50.6 ± 14.6 years; range, 20–74 years) were randomly selected by a 5-year age strata and gender. Of these, 3486 were eligible for this study. Among the eligible patients, 1700 (48.8% of the eligible subjects; 821 men and 879 women; mean age, 52.2 ± 14.3 years) underwent clinical examinations. At the time of peripheral blood collection, all cases and control subjects provided informed consent to participate in this study.

The NHL cases for this study were provided by the Chonnam National University Hwasun Hospital National Biobank of Korea, a member of the National Biobank of Korea, which is supported by the Ministry of Health, Welfare and Family Affairs. This study was approved by the Institutional Review Board of the Chonnam National University Hwasun Hospital in Hwasun, Korea.

### 4.2. Genotyping

Genomic DNA was extracted from peripheral blood using a QIAamp DNA Blood Mini Kit (Qiagen, Valencia, CA, USA), according to the manufacturer’s protocol. Genotyping to analyze polymorphisms was performed using polymerase chain reaction (PCR)-restriction fragment length polymorphism (RFLP) analysis, Simplex Pyrosequencing assays, high-resolution melt (HRM) analysis, and electrophoresis methods. Table 1 shows the primer sequences, methods, and references applied in this study. The Simplex Pyrosequencing assay was performed as previously described [8]. Specific primers and the sequencing primer were designed using Pyrosequencing SNP primer design software (v1.0.6, Biotage, Uppsala, Sweden). HRM genotyping was performed in 10-μL reaction volumes with 200 nM PCR primer, 1 μM Syto 9 fluorescent dye, 0.5 U f-Taq polymerase, and 40 ng of genomic DNA, using a Rotor-Gene 6000 high-resolution melter (Corbett Research, Sydney, Australia). RFLP genotyping was performed using the appropriate primers and restriction enzymes (Table 1).

Quality control (QC) for each genotyping, 96 samples of each genotyping assay were identified in 100% concordance by sequencing and 40 replicate samples were repeated with 100% compliance.

### 4.3. Statistical Analysis

The statistical significance of differences between the patient and control groups was estimated by logistic regression analysis. Control group was compared with DLBCL and T-cell lymphoma as well as NHL separately. The expected frequency of control genotypes was evaluated using the Hardy-Weinberg equilibrium test. Adjusted odds ratios (ORs) and 95% confidence intervals (CIs) were calculated using logistic regression models with adjustments for age and gender, to estimate the association between each genotype and NHL. Subjects with wild-type genotypes were considered to represent the baseline risk. Haplotype frequencies for *ABCB1* were evaluated using PHASE v2.1 software based on the Bayesian inference algorithm (http://stephenslab.uchicago.edu/software.html) [56]. Analyses were performed using SPSS software version 11.0 (SPSS, Chicago, IL, USA).

## 5. Conclusions

In the present study, we investigated the *XRCC1* 399 GA, *OGG1* 326 GG, *BRCA1* 871 TT, *WRN* 787 TT, and the *ABCB1* 1236 CC genotypes were associated with a decreased risk for NHL. Conversely, the *MGMT* 115 CT and *ABCB1* 3435 CT and TT genotypes were associated with an increased risk for NHL. Our results suggest that polymorphisms in the DNA repair genes *XRCC1*, *OGG1*, *BRCA*, *WRN1*, and *MGMT* and in *MDR1* may affect the risk for NHL in Korean patients.

## Figures and Tables

**Table 1.** Primer sequences and genotyping methods.

Gene and rs No.	Genotype	Primers and probe	Method	Reference
**DNA repair pathway**				

*ERCC1* rs3212961	A/C, IVS5 +33	F: TTGTCCAGGTGGATGTGGTA R: CCTCGCTGAGGTTTTAGCTG	HRM	
*ERCC2*/*XPD* rs13181	G/T, Lys751Gln	F: TCAAACATCCTGTCCCTACT R: CTGCGATTAAAGGCTGTGGA	RFLP: PstI	
*XPC* rs2228001	A/C, Lys939Gln	F: CCTCAAAACCGAGAAGATGAAG R: CAGGTGTGGGGCCTGTAGT	HRM	
*XRCC3* rs861539	C/T, Thr241Met	F: CCATTCCGCTGTGAATTTG R: GAAGGCACTGCTCAGCTCAC	HRM	
*RAD51* rs1801321	G/T, 172 in 5′-UTR	F: GTAGAGAAGTGGAGCGTAAGCC R: Biotin-CTGCGCCTCACACACTCA S: GGGGGCCGTGCGGGT	PSQ	
*XRCC4* rs1056503	G/T, Ser307Ser	F: AGGCCTGATTCTTCACTACCTG R: GGCTGCTGTTTCTCAGAGTTTC	HRM	
*XRCC1* rs1799782	C/T, Arg194Trp	F: ATAATACTGACCTTGCGGGACC R: Biotin-ACCCACGAGTCTAGGTCTCAA S: CTGAGGCCGGGGGCT	PSQ	
*XRCC1* rs25487	G/A, Arg399Gln	F: TCTCCCTTGGTCTCCAACCT R: AGTAGTCTGCTGGCTCTGG	RFLP: HpaII	[31]
*OGG1* rs1052133	C/G, Ser326Cys	F: CCCTCCTACAGGTGCTGTTC R: TGGGGAATTTCTTTGTCCAG	HRM	
*MGMT* rs12917	C/T, Leu115Phe	F: Biotin-CCCCTGTTCTCACTTTTGCA R: ACTGTGATGTCAGCGATCGTTAAT S: AAACGGGATGGTGAA	PSQ	
*BRCA1* rs799917	C/T, Pro871Leu	F: CCACAGTCGGGAAACAAGCATAGA R: CTTCTGCATTTCCTGGATTTGAAACC	RFLP: HpaII	[32]
*WRN* rs1800392	G/T, Leu787Leu	F: TGGGAATTTGAAGGTCCAAC R: GCATGGTATGTTCCACAGGA	HRM	

**Multidrug resistance**				

*ABCB1* rs1128503	1236, T/C, Gly412Gly	F: TCTTTGTCAC TTTATCCAGC R: TCTCACCATC CCCTCTGT	RFLP: EcoO191I	[33]
*ABCB1* rs2032582	2677, G/T/A, Ser893Thr/Ala	F: TGCAGGCTATAGGTTCCAGG R: TTTAGTTTGACTCACCTTCCCG	RFLP: Ban1, Rsa1	[34]
*ABCB1* rs1045682	3435, C/T, Ile1145 Ile	F: GAGCCCATCCTGTTTGACTG R: AGAGAGGCTGCCACATGCT	RFLP: Mbo1	[34]

PSQ: Pyrosequencing; HRM: High-resolution melter; RFLP: Restriction fragment length polymorphism.

**Table 2.** Association between polymorphisms in DNA repair pathway and risk of non-Hodgkin lymphoma (NHL).

SNP §	Genotype/ Haplotype	Control *n* (%)	NHL			DLBCL			T-cell lymphoma		
		
			*n* (%)	*OR* * (95% CI)	*p*	*n* (%)	*OR* * (95% CI)	*p*	*n* (%)	*OR* * (95% CI)	*p*
*ERCC1*	AA	440 (26)	153 (23)	1		79 (22)	1		34 (25)	1	
IVS5 +33C>A	AC	863 (51)	356 (54)	1.20 (0.96–1.50)	0.12	193 (53)	1.26 (0.95–1.69)	0.11	72 (54)	1.08 (0.71–1.66)	0.71
rs3212961	CC	395 (23)	156 (23)	1.14 (0.88–1.48)	0.33	89 (25)	1.28 (0.91–1.79)	0.16	28 (21)	0.91 (0.54–1.54)	0.73
	AC/CC	1258 (74)	512 (77)	1.18 (0.95–1.46)	0.13	282 (78)	1.27 (0.96–1.67)	0.09	100 (75)	1.03 (0.69–1.54	0.89
*ERCC2/XPD*	GG	1516 (89)	622 (90)	1		342 (91)	1		127 (90)	1	
Lys751Gln	GT	179 (11)	65 (9)	0.91 (0.68–1.24)	0.56	31 (8)	0.81 (0.54–1.21)	0.31	12 (8)	0.79 (0.43–1.45)	0.44
rs13181	TT	5 (0)	6 (1)	3.22 (0.98–10.62)	0.06	4 (1)	3.67 (0.96–14.03)	0.06	1 (1)	2.30 (0.26–20.29)	0.45
	GT/TT	184 (11)	71 (10)	0.97 (0.73–1.31)	0.86	35 (9)	0.89 (0.61–1.31)	0.56	13 (9)	0.83 (0.46–1.50)	0.53
*XPC*	AA	650 (38)	271 (39)	1		143 (38)	1		61 (44)	1	
Lys939Gln	AC	812 (48)	341 (49)	1.02 (0.84–1.23)	0.87	186 (49)	1.05 (0.82–1.34)	0.70	61 (44)	0.80 (0.55–1.16)	0.24
rs2228001	CC	238 (14)	81 (12)	0.83 (0.62–1.11)	0.20	48 (13)	0.96 (0.67–1.39)	0.84	18 (12)	0.79 (0.46–1.37)	0.40
	AC/CC	1050 (62)	422 (62)	0.97 (0.81–1.17)	0.77	234 (62)	1.03 (0.82–1.30)	0.80	79 (56)	0.97 (0.81–1.17)	0.77
*XRCC3*	CC	1573 (93)	635 (92)	1		343 (92)	1		129 (93)	1	
Thr241Met	CT	122 (7)	53 (8)	1.14 (0.81–1.60)	0.46	31 (8)	1.27 (0.84–1.94)	0.26	10 (1)	1.04 (0.53–2.03)	0.91
rs861539	TT	2 (0)	1 (0.1)	1.23 (0.11–13.69)	0.87	0 (0)	0.00 (0.00–0.00)	0.99	0 (0)	0.00 (0.00–0.00)	0.99
	CT/TT	124 (7)	54 (8)	1.14 (0.81–1.59)	0.45	31 (8)	1.25 (0.82–1.90)	0.30	10 (1)	1.02 (0.52–2.00)	0.95
*RAD51*	GG	1569 (92)	651 (94)	1		351 (93)	1		132 (94)	1	
172 in 5′-UTR	GT	129 (8)	43 (6)	0.82 (0.57–1.17)	0.27	27 (7)	0.97 (0.63–1.51)	0.90	8 (6)	0.44 (0.36–1.56)	0.44
rs1801321	TT	2 (0)	0 (0)	NA		0 (0)	NA		0 (0)	NA	
	GT/TT	131 (8)	43 (6)	0.81 (0.56–1.16)	0.24	27 (7)	0.96 (0.62–1.49)	0.86	8 (6)	0.74 (0.35–1.54)	0.42
*XRCC4*	GG	916 (54)	391 (56)	1		214 (57)	1		77 (55)	1	
Ser307Ser	GT	658 (39)	242 (35)	0.87 (0.72–1.05)	0.15	129 (34)	0.85 (0.67–1.08)	0.19	51 (36)	0.93 (0.64–1.35)	0.71
rs1056503	TT	124 (7)	61 (9)	1.11 (0.80–1.55)	0.53	35 (9)	1.17 (0.78–1.77)	0.45	12 (9)	1.09 (0.57–2.06)	0.80
	GT/TT	782 (46)	303 (44)	0.91 (0.76–1.09)	0.30	164 (43)	0.90 (0.72–1.13)	0.37	63 (45)	0.96 (0.68–1.36)	0.81
*XRCC1*	CC	776 (46)	294 (43)	1		156 (42)	1		63 (45)	1	
Arg194Trp	CT	741 (44)	318 (46)	1.13 (0.93–1.36)	0.21	176 (47)	1.17 (0.92–1.50)	0.19	63 (45)	1.05 (0.73–1.51)	0.81
rs1799782	TT	164 (10)	77 (11)	1.22 (0.90–1.66)	0.20	43 (11)	1.28 (0.87–1.87)	0.21	14 (10)	1.05 (0.57–1.92)	0.87
	CT/TT	905 (54)	395 (57)	1.15 (0.96–1.38)	0.13	219 (58)	1.19 (0.95–1.50)	0.13	77 (55)	1.05 (0.74–1.48)	0.80
*XRCC1*	GG	914 (54)	410 (59)	1		223 (59)	1		80 (57)	1	
Arg399Gln	GA	693 (41)	247 (36)	0.80 (0.66–0.96)	0.02	136 (36)	0. 82 (0.65–1.04)	0.10	51 (37)	0. 83 (0.57–1.20)	0.31
rs25487	AA	91 (5)	36 (5)	0.92 (0.61–1.38)	0.67	18 (5)	0.83 (0.49–1.42)	0.50	9 (6)	1.15 (0.56–2.38)	0.70
	GA/AA	784 (46)	383 (41)	0.81 (0.68–0.97)	0.02	154 (41)	0.82 (0.65–1.03)	0.09	60 (43)	0.87 (0.61–1.23)	0.42
*OGG1*	CC	472 (28)	226 (33)	1		126 (33)	1		49 (35)	1	
Ser326CyS	CG	863 (51)	339 (49)	0.81 (0.66–1.00)	0.048	184 (49)	0.79 (0.61–1.02)	0.07	68 (49)	0.76 (0.52–1.12)	0.16
rs1052133	GG	365 (21)	123 (18)	0.70 (0.54–0.91)	0.008	67 (18)	0.68 (0.49–0.95)	0.02	22 (16)	0.59 (0.35–0.99)	0.04
	CG/GG	1228 (72)	462 (67)	0.78 (0.64–0.95)	0.01	251 (67)	0.75 (0.59–0.96)	0.02	90 (65)	0.71 (0.49–1.02)	0.06
*MGMT*	CC	1327 (78)	517 (75)	1		276 (73)	1		103 (74)	1	
Leu115Phe	CT	350 (21)	167 (24)	1.25 (1.01–1.54)	0.04	98 (26)	1.37 (1.05–1.79)	0.02	32 (23)	1.22 (0.80–1.85)	0.35
rs12917	TT	23 (1)	9 (1)	1.02 (0.46–2.24)	0.97	4 (1)	0.87 (0.29–2.58)	0.80	4 (3)	2.53 (0.85–7.51)	0.10
	CT/TT	373 (22)	176 (25)	1.23 (1.00–1.52)	0.05	176 (25)	1.34 (1.04–1.74)	0.03	36 (26)	1.29 (0.87–1.93)	0.21
*BRCA1*	CC	828 (49)	364 (53)	1		207 (55)	1		75 (54)	1	
Pro871Leu	CT	715 (42)	273 (40)	0.87 (0.72–1.05)	0.13	145 (38)	0.81 (0.64–1.03)	0.08	57 (41)	0.90 (0.63–1.29)	0.56
rs799917	TT	157 (9)	50 (7)	0.71 (0.50–0.997)	0.048	25 (7)	0.62 (0.39–0.97)	0.04	6 (4)	0.42 (0.18–0.97)	0.04
	CT/TT	872 (51)	323 (47)	0.84 (0.70–1.00)	0.05	170 (45)	0.77 (0.62–0.97)	0.03	63 (45)	0.81 (0.57–1.15)	0.24
*WRN*	GG	558 (33)	245 (36)	1		133 (35)	1		48 (34)	1	
Leu787Leu	GT	846 (50)	359 (52)	0.98 (0.80–1.19)	0.81	196 (52)	1.00 (0.78–1.29)	0.99	76 (55)	1.03 (0.71–1.51)	0.86
rs1800392	TT	296 (17)	85 (12)	0.68 (0.51–0.91)	0.01	48 (13)	0.73 (0.50–1.05)	0.09	15 (11)	0.60 (0.33–1.09)	0.09
	GT/TT	1142 (67)	444 (64)	0.90 (0.75–1.09)	0.28	244 (65)	0.93 (0.73–1.18)	0.56	91 (66)	0.80 (0.56–1.13)	0.21

§ SNP; single nucleotide polymorphism;

* ORs and 95% CIs were estimated using multiple logistic regression and adjusted for sex and age.

**Table 3.** Associations between polymorphisms in *MDR1* and the risk for NHL.

SNP	Genotype/Haplotype	Control *n* (%)	NHL			DLBCL			T-cell lymphoma		
		
			*n* (%)	*OR* * (95% CI)	*p*	*n* (%)	*OR* * (95% CI)	*p*	*n* (%)	*OR* * (95% CI)	*p*
*ABCB1* 1236	TT	580 (34)	234 (34)	1		139 (37)	1		44 (32)	1	
Gly412Gly	TC	825 (49)	367 (53)	1.11 (0.91–1.36)	0.29	193 (51)	1.00 (0.78–1.28)	0.98	74 (53)	1.18 (0.80–1.74)	0.41
rs1128503	CC	295 (17)	87 (13)	0.74 (0.56–0.98)	0.04	44 (12)	0.64 (0.44–0.93)	0.02	21 (15)	0.93 (0.54–1.60)	0.80
	TT/CC	1120 (66)	454 (66)	1.01 (0.84–1.22)	0.89	237 (63)	0.90 (0.71–1.14)	0.39	95 (68)	1.11 (0.77–1.62)	0.57
*ABCB1* 2677	GG	323 (19)	131 (19)	1		64 (17)	1		29 (21)	1	
Ser893 Thr/Ala	GW †	886 (52)	340 (50)	0.96 (0.75–1.22)	0.74	189 (51)	1.10 (0.81–1.51)	0.54	69 (50)	0.86 (0.55–1.35)	0.51
rs2032582	WW	488 (29)	210 (31)	1.07 (0.82–1.39)	0.61	115 (31)	1.21 (0.86–1.71)	0.27	41 (29)	0.92 (0.56–1.51)	0.74
	GW/WW	1374 (81)	550 (81)	1.00 (0.80–1.26)	0.99	304 (82)	1.14 (0.85–1.54)	0.39	110 (79)	0.88 (0.57–1.35)	0.56
*ABCB1* 3435	CC	708 (42)	234 (34)	1		128 (34)	1		46 (33)	1	
Ile1145 Ile	CT	800 (47)	377 (54)	1.50 (1.23–1.82)	<0.0001	210 (56)	1.52 (1.19–1.94)	0.01	76 (54)	1.51 (1.04–2.22)	0.03
rs1045642	TT	192 (11)	83 (12)	1.43 (1.06–1.93)	0.02	40 (10)	1.25 (0.84–1.86)	0.27	18 (13)	1.54 (0.87–2.72)	0.14
	CT/TT	992 (58)	460 (66)	1.48 (1.23–1.79)	<0.0001	250 (66)	1.47 (1.16–1.86)	0.02	94 (67)	1.52 (1.05–2.19)	0.03
*ABCB1*	TTT	1037 (31)	444 (34)	1		242 (34)	1		88 (34)	1	
HAP	CGC	734 (22)	261 (20)	0.83 (0.69–0.995)	0.04	132 (19)	0.77 (0.61–0.98)	0.03	53 (20)	0.85 (0.60–1.21)	0.38
	TGC	713 (21)	270 (21)	0.88 (0.73–1.05)	0.15	152 (22)	0.90 (0.71–1.13)	0.35	54 (21)	0.91 (0.64–1.29)	0.59
	CAC	583 (18)	211 (16)	0.84 (0.69–1.02)	0.08	117 (17)	0.87 (0.68–1.11)	0.25	44 (17)	0.89 (0.61–1.29)	0.53
	TTC	186 (6)	70 (6)	0.90 (0.67–1.21)	0.48	45 (6)	1.07 (0.75–1.53)	0.72	11 (4)	0.73 (0.38–1.39)	0.34
	CGT	62 (2)	41 (3)	1.67 (1.10–2.54)	0.02	16 (2)	1.22 (0.68–2.18)	0.51	12 (4)	2.48 (1.28–4.79)	0.007

* ORs and 95% CIs were estimated using multiple logistic regression and adjusted for sex and age;

† W indicated all alleles except G allele.

GW: GT + GA, WW: TT + TA + AA.
